# Kalman filtering to reduce measurement noise of sample entropy: An electroencephalographic study

**DOI:** 10.1371/journal.pone.0305872

**Published:** 2024-07-29

**Authors:** Nan Zhang, Yawen Zhai, Yan Li, Jiayu Zhou, Mingming Zhai, Chi Tang, Kangning Xie

**Affiliations:** 1 School of Biomedical Engineering, Air Force Medical University, Xi’an, China; 2 Shaanxi Provincial Key Laboratory of Bioelectromagnetic Detection and Intelligent Perception, Xi’an, China; Polytechnic University of Marche: Universita Politecnica delle Marche, ITALY

## Abstract

In the analysis of electroencephalography (EEG), entropy can be used to quantify the rate of generation of new information. Entropy has long been known to suffer from variance that arises from its calculation. From a sensor’s perspective, calculation of entropy from a period of EEG recording can be treated as physical measurement, which suffers from measurement noise. We showed the feasibility of using Kalman filtering to reduce the variance of entropy for simulated signals as well as real-world EEG recordings. In addition, we also manifested that Kalman filtering was less time-consuming than moving average, and had better performance than moving average and exponentially weighted moving average. In conclusion, we have treated entropy as a physical measure and successfully applied the conventional Kalman filtering with fixed hyperparameters. Kalman filtering is expected to be used to reduce measurement noise when continuous entropy estimation (for example anaesthesia monitoring) is essential with high accuracy and low time-consumption.

## Introduction

Electroencephalography (EEG) is highly nonlinear and entropy measures have long been used in clinical practice to reveal the nonlinear nature, for example, in classifying walking limitations [1], analyzing complexity and variability of trunk accelerations in patients with Parkinson’s Disease [2, 3], differentiating balance patterns in diabetic patients with and without neuropathy [4], assessing anesthetic drug effects on the brain [5], identifying fetal distress [6], autism spectrum disorder in children [7], tinnitus [8], attention deficit hyperactivity disorder [9], epilepsy [10], Alzheimer’s disease [11], schizotypy [12], mind wandering [13] and psychogenic non-epileptic seizures [14]. Examples of entropy measures are permutation entropy [15], approximate entropy [16], neural network entropy [17], dispersion entropy [18], sample entropy [19, 20] and their variants [21–25].

Most studies on the entropy measures of EEG focus on the values of entropy at some specific moments while there exist some circumstances that emphasize the evolving pattern. For example, Liang et al. use entropy measures to monitor depth of anesthesia [5]. Kbah et al. use entropy-based biomarkers to monitor epileptic EEG activity [10]. Díaz et al. show entropy dynamic map of EEG in resting conditions [26].

Calculation of entropy can be conceived as a physical sensor to measure the irregularity of the time series in question. Like any other measure of sensors [27], sample entropy also suffers from the inherent measurement noise, which can be estimated numerically [28]. The measurement noise of entropy should be reduced to achieve more accurate estimation. Conventional smoothing methods, moving average [29], exponentially weighted moving average (EWMA) [30] can be applied to the continuously computed (measured) entropy values with a sliding window. The two methods may suffer from high computational cost or low performance, which may not be optimal when online monitoring of entropy measures is required.

As a powerful technology for estimating the states of a dynamic system, the Kalman filtering is usually applied to the recorded time series, e.g., EEG [31], electrocardiogram [32], positioning in global positioning system [33] and drone tracking [34].

In this paper, we propose a measurement noise-reducing method for entropy, in which, the Kalman filtering operates on the continuous calculated entropy values of EEG time series with non-overlapping sliding windows. We test this method on simulated signals (power noise, Logistic map signals and Rössler system signals) as well as EEG recordings from publicly available datasets (sleep EEG, and EEG recordings from pediatric subjects with refractory seizures). We also compare the smoothing effects and computational costs among three smoothing methods. We also study the effects of hyperparameters of Kalman filtering on the variance reduction.

## Materials and methods

### Datasets

#### Simulation signal generation

First, we generated power-law time series (also called power noise) with power spectrum of 1/*f*^*β*^ following [35]. The signals were generated with known *β*: 0, 0.5, 1, representing white noise, pink noise and 1/f noise respectively. Each time series contains 50,000 data points at a sampling rate of 100 Hz. The power noise signals were generated using the Matlab toolbox *powernoise.m* provided in [36].

Logistic map signals are often used [6] to compare entropy measures to the original work by Costa et al [37]. Logistic map signal can be defined by

$$\begin{array}{c} x_{i+1}=r\left(1-x_{i}\right) \end{array}$$ (1)

We generated logistic map signals using parameters *r* = 3.57, 3.77 and 3.9.

Rössler system signals can also be used as a test dataset to assess entropy related properties [35]. A Rössler system is expressed as [38]

$$\begin{array}{c} \left\{ \begin{array}{cc} \frac{dx}{dt}=-y-z & \\ \frac{dy}{dt}=x+ay & \\ \frac{dz}{dt}=b+z\left(x-c\right) \end{array}\right. \end{array}$$ (2)

We generated Rössler system time seires using parameters *a* = 0.38, *b* = 0.2, *c* = 5.7 following [35] and two additional parameters *c* = 2.5 and 4.

#### Real-world dataset

In this study, two EEG datasets were analyzed. The first EEG dataset is the Sleep-EDF Expanded Database open sleep dataset published on physioNet. One hundred and fifty three SC* files (SC, sleep cassette) were obtained in a 1987-1991 study on the age effects [39, 40]. Polysomnograms were recorded twice, for approximately 20 hours each time, at a sampling rate of 100 Hz. Polysomnograms contained Fpz-Cz- and Pz-Oz-based EEG signals, horizontal electrooculogram signals, sub-chin electromyography, and event markers. All polysomnograms were manually scored by trained technicians according to the 1968 Rechtschaffen and Kales manual. Polysomnograms included sleep stages ‘W’, ‘R’, ‘1’, ‘2’, ‘3’, ‘4’, ‘M’ and ‘?’, representing Wake, REM, S1, S2, S3, S4, movement and unlabeled, respectively. In our study, in order to investigate how Kalman filtering can reduce the variance of entropy on slower changing sleep data, we chose to analyze the same channels in different people. The window length for entropy calculation is 5 times the sampling frequency (100 Hz), i.e., 500.

Our second dataset was derived from the CHB-MIT Scalp EEG Database, a collection of EEG recordings from 22 (5 males, 17 females) pediatric subjects with refractory seizures collected by Children’s Hospital Boston [41, 42]. Detailed descriptions of these samples are on the physioNet website. The subjects were monitored for up to several days after discontinuation of antiepileptic drugs. Continuous EEG data were recorded for each subject. A total of 182 seizure onset and end times were recorded. All data were acquired at a sampling rate of 256 Hz with 16-bit resolution. These recordings were made using the International 10-20 EEG Electrode Location and Naming System. In this study, in order to investigate the performance of Kalman filtering in reducing the variance of entropy for transient EEG activities, we chose to analyze EEG segments of one-hour that contain seizures from channels FP1-F3, F3-C3, C3-P3 and P3-O1 in the same subject (chb03). The window length for entropy calculation is 5 times the sampling frequency (256 Hz), i.e., 1280.

### Sample entropy

Sample entropy is invented by Richman et al [43] and is briefly summarized here to show the principles and parameter choice. Let the raw data sampled at equal event intervals be *u*(*i*), *i* = 1, 2, ⋯, *N*. First, Reconstructing m-dimensional vectors *x*(1), *x*(2), ⋯ *x*(*N* − *M* + 1), where

$$\begin{array}{c} x(i)=[u(i),u(i+1),\cdots u(i+m-1)],i=1∽N-m+1 \end{array}$$ (3)

Define the distance *d*[*x*(*i*), *x*(*j*)] between *x*(*i*) and *x*(*j*) to be the one with the largest difference between the corresponding elements of the two, i.e.,

$$\begin{array}{c} d=[x(i),x(j)]=\underset{a}{\text{max}}|u(a)-\overset{*}{u(a)}|,i\neq j \end{array}$$ (4)

where *u*(*a*) is an element in the vector x. For each value of i calculate the distance *d*[*x*(*i*), *x*(*j*)] between *x*(*i*) and the other vectors *x*(*j*), j = 1, 2, ⋯, *N* − *m* + 1.

According to the given threshold *r*(*r* > 0), for 1 ⩽ *i* ⩽ *N* − *m* + 1, the number of *d*[*x*(*i*), *x*(*j*)] < *r* is counted for each value of i and the ratio to the total number of vectors *N* − *m* is denoted as $B_{i}^{m}(m)$.

$$\begin{array}{c} B_{i}^{m}(m)=\frac{num\{d[x(i),x(j)]<r\}}{N-m},i\neq j \end{array}$$ (5)

The average of the $B_{i}^{m}(m)$ over all values of *i*, denoted *B*^*m*^(*r*), i.e.,

$$\begin{array}{c} B^{m}(r)=\frac{1}{N-m+1}\sum_{i=1}^{N-m+1}B_{i}^{m}(r) \end{array}$$ (6)

Then increase the dimension *m* to *m* + 1 to get $B_{i}^{m+1}(r)$. Thus the sample entropy is defined as

$$\begin{array}{c} \text{SampEn}(m,n)=\underset{N\to \infty }{\text{lim}}\{-\text{ln}\frac{B^{m+1}(r)}{B_{\left(r\right)}^{m}}\} \end{array}$$ (7)

Since *N* cannot be ∞,

$$\begin{array}{c} \text{SampEn}(m,r,N)=-\text{ln}[\frac{B^{m+1}(r)}{B_{\left(r\right)}^{m}}] \end{array}$$ (8)

where *N* is the length of the data; *m* is the embedding dimension, *r* is the threshold (is calculated as *r* = *c* ⋅ *σ*, where, *σ* is the standard deviation of the original sequence).

The conditional probability (CP) is defined as

$$\begin{array}{c} \text{CP}=\frac{A}{B}=\frac{B^{m+1}(r)}{B_{\left(r\right)}^{m}} \end{array}$$ (9)

There is no a priori parameter setting precedure currently. According to the evaluation by Lake et al. [28] for the neonatal heart rate data, *m* should be 2 or 3; *c* is between 0.1 and 0.25. The above choice of parameters is widely accepted [35, 44, 45]. In this study, we adopted typical values of embedding dimension *m* and threshold *r*, i.e., *m* = 2, *r* = 0.15*σ*. We also compared the effects of different choices of *m* (*m* = 2 or 3) and *r* (in the range of 0.1*σ* and 0.25*σ*).

#### Theoretical estimation of sample entropy variance

The theoretical variance of sample entropy can be numerically calculated [28] as

$$\begin{array}{c} \sigma_{CP}^{2}=\frac{CP(1-CP)}{B}+\frac{1}{B^{2}}[K_{A}-K_{B}({CP}^{2})] \end{array}$$ (10)

where *K*_*A*_ is the number of pairs of matching templates of length *m* + 1 that overlap and *K*_*B*_ is the number of pairs of matching templates of length *m* that overlap.

### Other entropy measures

To test the feasibility of the proposed Kalman filtering, we also tried two additional entropy measures.

#### Approximate entropy

Approximate entropy is introduced by Pincus et al [16] and is briefly summarized here to show the principles and parameter choice. Let the raw data sampled at equal event intervals be *u*(*i*), *i* = 1, 2, ⋯, *N*. First, Reconstructing m-dimensional vectors *x*(1), *x*(2), ⋯ *x*(*N* − *M* + 1), where

$$\begin{array}{c} x(i)=[u(i),u(i+1),\cdots u(i+m-1)],i=1∽N-m+1 \end{array}$$ (11)

Define the distance *d*[*x*(*i*), *x*(*j*)] between *x*(*i*) and *x*(*j*) to be the one with the largest difference between the corresponding elements of the two, i.e.,

$$\begin{array}{c} d=[x(i),x(j)]=\underset{a}{\text{max}}|u(a)-\overset{*}{u(a)}|,i\neq j \end{array}$$ (12)

where *u*(*a*) is an element in the vector x. For each value of i calculate the distance *d*[*x*(*i*), *x*(*j*)] between *x*(*i*) and the other vectors *x*(*j*), j = 1, 2, ⋯, *N* − *m* + 1.

According to the given threshold *r*(*r* > 0), for 1 ⩽ *i* ⩽ *N* − *m* + 1, the number of *d*[*x*(*i*), *x*(*j*)] < *r* is counted for each value of i and the ratio to the total number of vectors *N* − *m* + 1 is denoted as $B_{i}^{m}(m)$.

$$\begin{array}{c} B_{i}^{m}(m)=\frac{num\{d[x(i),x(j)]<r\}}{N-m+1} \end{array}$$ (13)

Take the logarithm of $B_{i}^{m}(m)$ first, and then find its average over all *i*.denoted as,

$$\begin{array}{c} \Phi^{m}(r)=\frac{\sum_{i=1}^{N-m+1}\text{ln}\;B_{i}^{m}(r)}{N-m+1} \end{array}$$ (14)

Then increase the dimension *m* to *m* + 1 to get Φ^*m*+1^(*r*). Thus the approximate entropy is defined as

$$\begin{array}{c} \text{ApEn}(m,r,N)=\Phi^{m}(r)-\Phi^{m+1}(r) \end{array}$$ (15)

where *N* is the length of the data; *m* is the embedding dimension, *r* is the threshold.

There is no a priori parameter setting procedure currently. According to the evaluation by Bajić et al [46], *m* should be 2 or 3; *r* is between 0.1*σ* and 0.25*σ* (*σ* is the standard deviation of the time series *u*(*i*)). In this study, we adopted typical values of embedding dimension *m* = 2 and threshold *r* = 0.15*σ*.

#### Neural network entropy

Recently, a new entropy measure has been proposed, namely, neural network entropy (NNetEn), which is a neural network-based technique for entropy estimation of time series data [17]. Unlike conventional entropy measures, NNetEn does not consider a probability distribution, and depends on only one parameter, number of epochs in the LogNNet model [47]. We used Method 1 (Row-wise filling) with duplication and set epoch to 20 to calculate the NNetEn entropy.

### Kalman filtering

Kalman filtering is an algorithm that utilizes the state equation of a linear system to optimally estimate and predict the state of the system by means of the input and output observations of the system [48]. Since the observation data include the influence of noise and interference in the system, the optimal estimation can also be regarded as a filtering (smoothing) process. The principle is to use the Kalman gain to correct the state prediction value to make it close to the real value. It consists of two main steps: prediction and update. The algorithm is divided into the following steps:

A system is represented by a discrete state-space equation as

$$\begin{array}{c} \left\{ \begin{array}{c} X_{k}=\text{A}X_{k-1}+\text{B}U_{k-1}+W_{k-1}\\ Z_{k}=\text{H}X_{k}+V_{k} \end{array}\right. \end{array}$$ (16)

where *X*_*k*_ is the state variable, the “idea” entropy which is free from variance interference; *Z*_*k*_ is the measurement variable, which is normally calculated from an algorithm (e.g., sample entropy, approximate entropy, NNetEn entropy). *W*_*k*−1_ and *V*_*k*_ are the noises in the system and in the measurement process, which obey normal distributions with zero mean and covariance matrices **Q**, **R**, respectively, i.e., *W*_*k*_ ∽ *P*(0, **Q**), *V*_*k*_ ∽ *P*(0, **R**). *U*_*k*_ is the control variable that relates the optional control input to the state variable *X*_*k*_. *A* is the state transfer matrix that relates the state at the previous time step to the state at the current step. **B** is the control matrix that relates the optional control input to the state *X*_*k*_. **H** is the state observation matrix, which relates the state to the measurements.

The Kalman algorithm is divided into two steps, prediction and update.

Prediction: estimate the state at the current moment, namely moment *k*, based on the a posteriori estimate ${\hat{X}}_{k-1}$ of the previous moment *k* − 1, and get the a priori estimate ${\hat{X}}_{k}^{-}$ of moment *k*; that is

$$\begin{array}{c} {\hat{X}}_{k}^{-}=\text{A}{\hat{X}}_{k-1}+\text{B}U_{k-1} \end{array}$$ (17)

Based on the covariance matrix **P**_*k*−1_ of the error *e*_*k*−1_ at moment *k* − 1 and the covariance matrix **Q** of the process noise *w*, the covariance matrix ${\text{P}}_{K}^{-}$ of the error *e*_*k*_ at moment *k* of the prediction is taken, which is.

$$\begin{array}{c} {\text{P}}_{K}^{-}=\text{A}{\text{P}}_{k-1}{\text{A}}^{T}+\text{Q} \end{array}$$ (18)

Update: Correct the prediction stage estimates using the current moment measurements to get the current moment a posteriori estimates.

Calculate the Kalman gain coefficient *V*_*k*_ at moment *k*.that is

$$\begin{array}{c} K_{k}=\frac{{\text{P}}_{k}^{-}{\text{H}}^{T}}{\text{H}{\text{P}}_{k}^{-}{\text{H}}^{T}+\text{R}} \end{array}$$ (19)

Corrective updating of the state using the Kalman gain yields the estimate ${\hat{X}}_{k}$ at moment *k*, i.e.

$$\begin{array}{c} {\hat{X}}_{k}={\hat{X}}_{k}^{-}+K_{k}(Z_{k}-\text{H}{\hat{X}}_{k}^{-}) \end{array}$$ (20)

The iteration that estimates the optimal value at the *k* + 1 moment performs the update operation. That is

$$\begin{array}{c} {\text{P}}_{k}=(I-K_{k}\text{H}){\text{P}}_{k}^{-} \end{array}$$ (21)

where **I** is the identity matrix.

In this study, since we are dealing with scalar variables, the matrix **A**, **B**, **H**, **P**, etc can then be expressed as scalars, *A*, *B*, *H*, *P*, etc. Similarly, covariance matrices **Q**, **R** become variance of system *Q*, and variance of measurement *R*. *A* and *H* are set to 1; the control variable *U*_*k*_ is set to 0. Therefore, the linear dynamic system can be described by state equation

$$\begin{array}{c} \left\{ \begin{array}{c} X_{k}=X_{k-1}+W_{k-1}\\ Z_{k}=X_{k}+V_{k} \end{array}\right. \end{array}$$ (22)

And the corresponding Kalman filtering process can be described as

$$\begin{array}{c} \left\{ \begin{array}{c} {\hat{X}}_{k}^{-}={\hat{X}}_{k-1}\\ P_{K}^{-}=P_{k-1}+Q\\ K_{k}=\frac{P_{k}^{-}}{P_{k}^{-}+R}\\ {\hat{X}}_{k}={\hat{X}}_{k}^{-}+K_{k}(Z_{k}-{\hat{X}}_{k}^{-})\\ P_{k}=(1-K_{k})P_{k}^{-} \end{array}\right. \end{array}$$ (23)

We set *X*_0_ to *Z*_0_, *Q* to 0.1, and *R* to 0.5.

### Benchmark smoothing methods

#### Moving average

Moving average is a classical filtering algorithm, whose main idea is to process the signal with a sliding window, in which the data are averaged. Moving average can reduce the variance of the signal by eliminating the periodic noise [49].

In this study, we apply the moving average algorithm to sample entropy values. The specific steps are as follows:

- Define the size of each window, i.e. the number of entropy points contained; the window size is defined as 5.
- Move the window to the next position from the starting point of the entropy values.
- Calculate the mean value of the entropy values within each window.
- Update entropy values. Use the calculated mean value to represent the entropy values within the window.

A series of smoothed entropy values can be obtained by constantly moving the window and calculating the mean values. These smoothed entropy values reduce the measurement noise of entropy.

#### Exponentially weighted moving average

Exponentially weighted moving average (EWMA) is a commonly used time series data smoothing technique [30]. The core idea of EWMA is that it gives a higher weight to the nearest data point, while its weight decreases exponentially as the data point gets further away from the current time point. The calculation formula is as follows [30]

- Initialize the first EWMA value as the first data point.
- For each time point *t*, the EWMA value is calculated according to the following formula:
  $$\begin{array}{c} \text{EMA}(t)=\alpha \times x(t)+(1-\alpha )\times \text{EMA}(t-1) \end{array}$$ (24)
  where EMA(*t*) represents the EWMA value at time point *t*, EMA(*t* − 1) represents the EWMA value at time point *t* − 1, *x*(*t*) represents the observed value at time point *t*, and *α* is the smoothing factor (usually a decimal number between (0,1)). The initial value of *α* is set to 1.
- The steps above are repeated until the EWMA values for all time points have been calculated.

### Variance reduction rate

We define the variance reduction rate (VRR) as

$$\begin{array}{c} \text{VRR}=\frac{V_{before}-V_{after}}{V_{before}}\times 100\% \end{array}$$ (25)

where *V*_*before*_ stands for the variance of the entire time series of sample entropy measure calculated over the EEG waveforms; and *V*_*after*_ stands for the variance of the entire time series of sample entropy measures that have been smoothed by Kalman filer.

### Statistical analysis

This distribution of entropy time series was tested using Shapiro-Wilk Normality test. One-way ANOVA was performed to examine if the significant differences existed between the four groups of thresholds *r* = 0.1*σ*, *r* = 0.15*σ*, *r* = 0.2*σ*, *r* = 0.25*σ*, as well as to examine if there are significant differences in VRR values for parameters *Q* and *P*. If there is a statistically significant difference between the means of groups, Bonferonni was used for post hoc multilpe comparisons.

## Results

### Simulations

**Fig 1:**
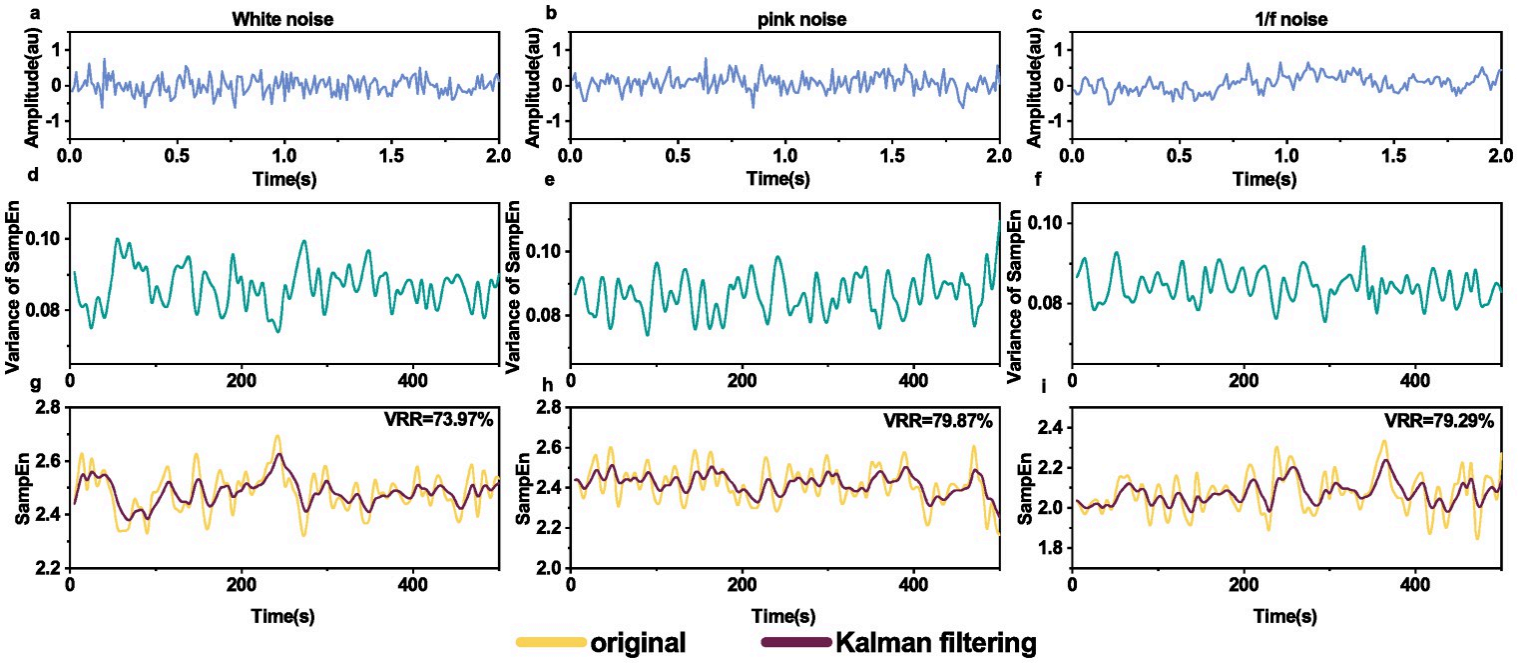
Kalman filtering on sample entropy time series of different power noise. The first row (**a-c**): the generated waveforms of power noise with different parameters, *β* = 0 (white noise), 0.5 (pink noise), and 1 (1/f noise). Segments of 2 seconds were shown for clarity. The second row (**d-f**): theoretical variances of sample entropy of the corresponding waveforms (**a-c**), which were calculated from Eq 10. The third row (**g-i**): the original and smoothed sample entropy time series of the corresponding waveforms (**a-c**) by Kalman filtering.

In the simulations, we applied the Kalman filtering to reduce measuring variance of entropy on power noise. We generated power noise on *β* = 0 (Fig 1a), *β* = 0.5 (Fig 1b), *β* = 1 (Fig 1c) for three examples. In order to quantify the differences before and after Kalman filtering, we first calculated three entropy time series. Then we calculated the theoretical variance in the three examples (Fig 1d–1f). Afterwards we applied Kalman filtering to reduce the measurement noise. Compared with the original entropy time series, VRR values of smoothed entropy time series by Kalman filtering were 73.97%, 79.87%, and 79.29% correspondingly.

**Fig 2:**
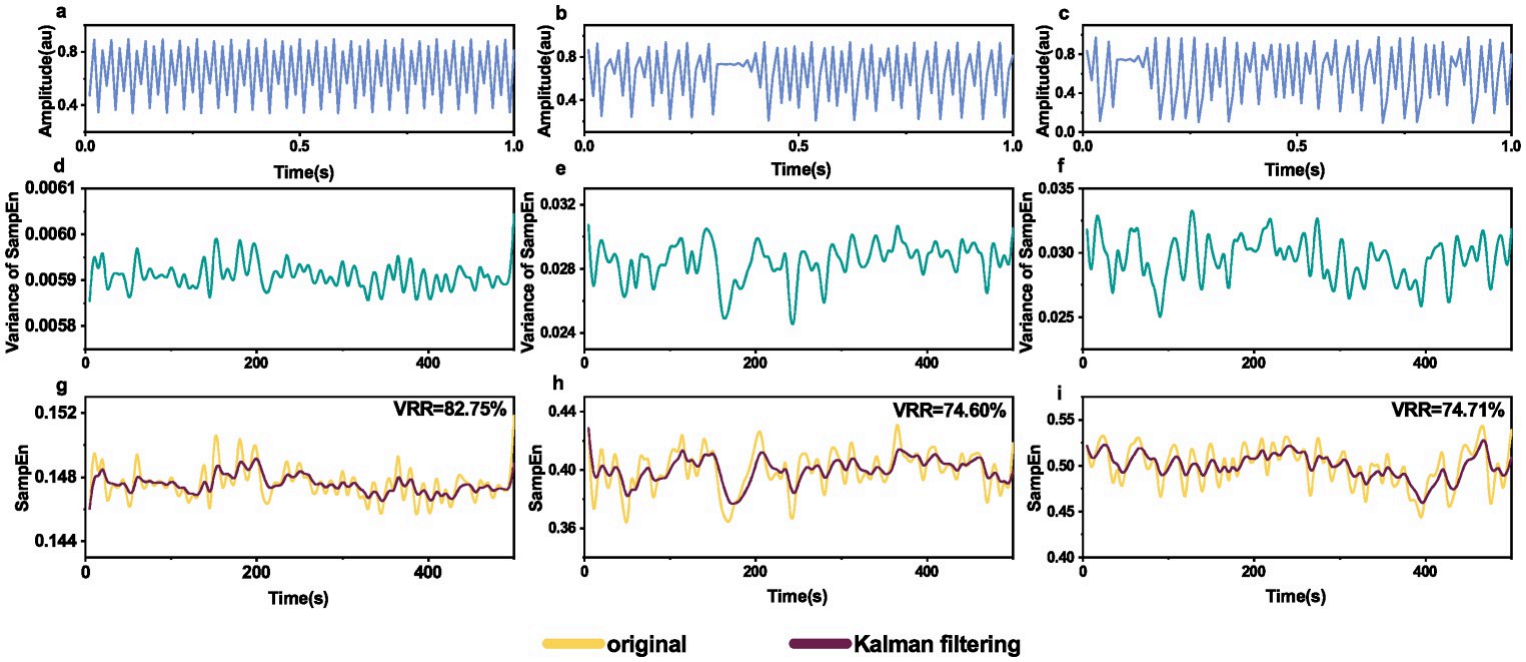
Kalman filtering on sample entropy time series of different logistic map signals. The first row (**a-c**): the generated waveforms of logistic map with different parameters, *r* = 3.57, 3.77, 3.9. The second row (**d-f**): the theoretical variance of sample entropy time series of the corresponding waveforms (**a-c**) calculated from Eq 10. The third row (**g-i**): the original and smoothed sample entropy time seires of the corresponding waveforms (**a-c**) by Kalman filtering.

Fig 2 shows the effects of Kalman filtering smoothing on different Logistic map signals. Compared with the original entropy time series, VRR values of smoothed entropy time series by Kalman filtering were 82.75%, 74.60%, and 74.71% correspondingly.

**Fig 3:**
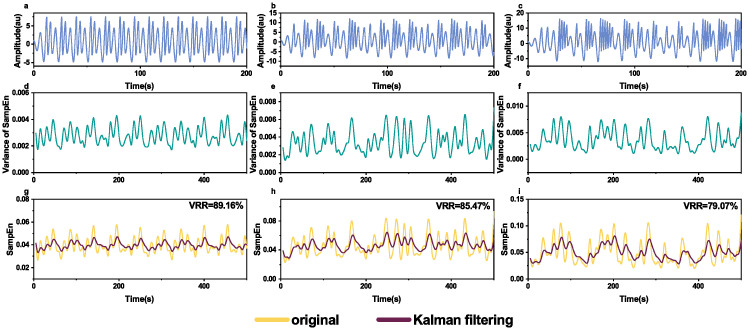
Kalman filtering on sample entropy time series of different Rössler system signals. The first row (**a-c**): the generated waveforms of logistic map with different parameters, *c* = 2.5, 4, 5.7. The second row (**d-f**): the theoretical variance of sample entropy time series of the corresponding waveforms (**a-c**) calculated from Eq 10. The third row (**g-i**): the original and smoothed sample entropy time seires of the corresponding waveforms (**a-c**) by Kalman filtering.

Similarly, Fig 3 shows the effects of Kalman filtering smoothing on different Rössler system signals. Compared with the original entropy time series, VRR values of smoothed entropy time series by Kalman filtering were 89.16%, 85.47%, and 79.07% correspondingly.

### EEG of sleep-EDF expanded database

**Fig 4:**
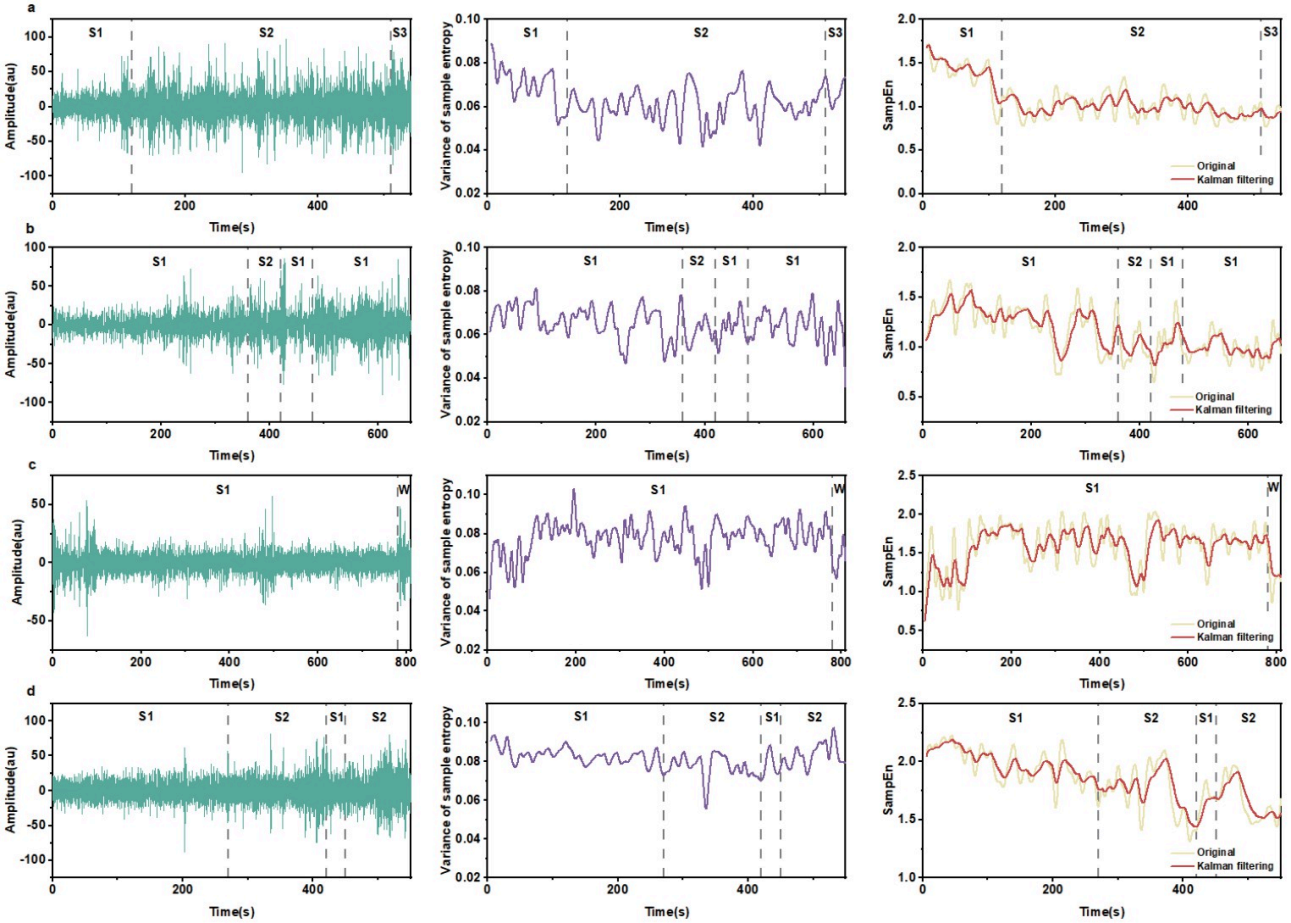
Kalman filtering on sample entropy time series of sleep signals. (**a-d**) demonstrate the smoothing effect on S1, S2, S3 and W stages for subjects SC4001E0, SC4011E0, SC4021E0, and SC4032E0. First column: EEG waveforms; second column: theoretical variance computed by Eq 10; third row: original sample entropy time series and the sample entropy time series after smoothing by Kalman filtering. S1: sleep stage 1; S2: sleep stage 2; S3: sleep stage 3; W: wake stage. From the third column, it is visually clear that Kalman filtering reduces the variance of sample entropy measures; detailed values, see Table 1.

We analyzed the signals from selected EEG data from sleep-EDF Database Expanded. Fig 4 shows four subjects with subject numbers: SC4001E0, SC4011E0, SC4021E0 and SC4032E0. A segment of EEG containing different stages of sleep was randomly selected for each subject. Stages S1, S2 and S3 for subject SC4001E0 are shown in Fig 4a. Stages S1 and S2 for subject SC4011E0 are shown in Fig 4b. Stages S1 and W for subject SC4021E0 are shown in Fig 4c. Two stages containing S1 and S2 for subject SC4032E0 are shown in Fig 4d. We calculated the theoretical variance of the four subjects according to Eq 10 as shown in the second column of Fig 4. We also calculated the sample entropy time series (marked as Original), and then applied Kalman filtering to produce the smoothed sample entropy time series (marked as Kalman filtering). Fig 4 visiually shows that Kalman filtering reduces the variance for all four subjects. VRR values by Kalman filtering were 33.16%, 51.06%, 52.73% and 39.14% for the four subjects (Table 1).

**Table 1:** Comparison of VRR by different methods for sleep data.

	SC4001E0	SC4011E0	SC4021E0	SC4032E0
SampEn	6.74E-02	7.53E-02	1.24E-01	6.21E-02
SampEn after MA	4.47E-02	4.80E-02	7.44E-02	4.16E-02
SampEn after EWMA	4.73E-02	4.41E-02	7.13E-02	4.29E-02
SampEn after KF	4.50E-02	3.68E-02	5.26E-02	3.78E-02
VRR by MA	33.71%	36.17%	39.96%	33.09%
VRR by EWMA	29.85%	41.38%	42.43%	30.92%
VRR by KF	33.16%	51.06%	52.73%	39.14%

Note: SampEn stands for sample entropy; MA stands for moving average; KF stands for Kalman filtering.

**Fig 5:**
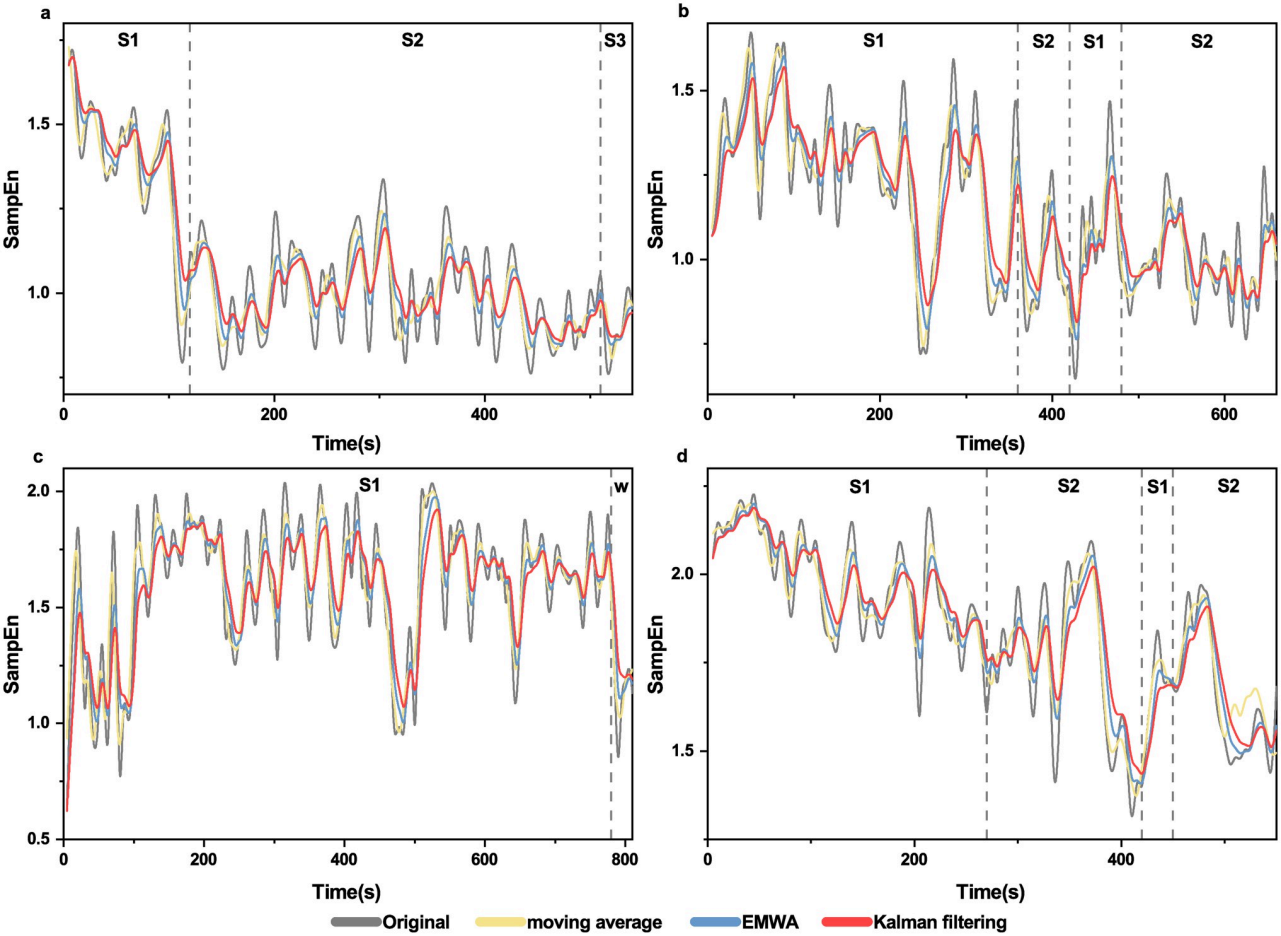
Visual comparison of three smoothing methods for sleep data. (**a**): subject SC4001E0, (**b**): subject SC4011E0, (**c**): subject SC4021E0, (**d**): subject SC4032E0. VRRs are shown in Table 1.

**Fig 6:**
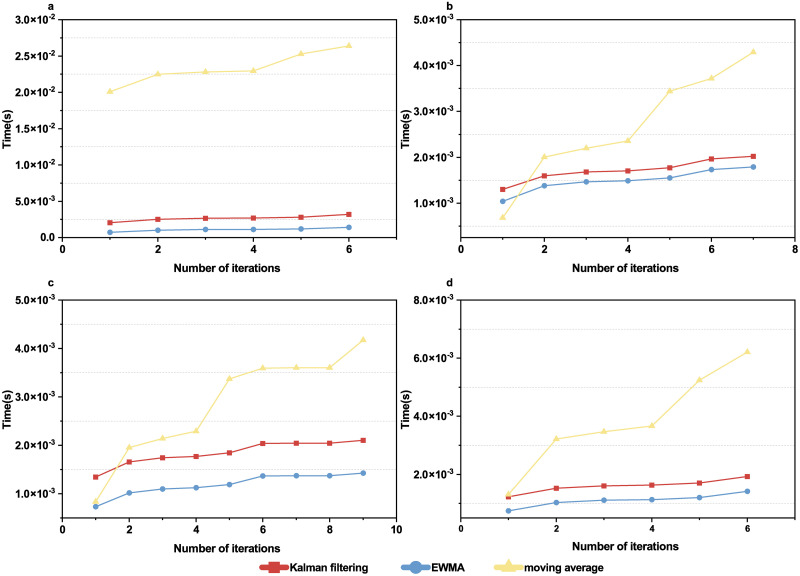
Comparison of cumulative computational time for sleep EEG data. Three smoothing methods for different subjects, as a function of iteration number. (**a**) subject SC4001E0, (**b**) subject SC4011E0, (**c**) subject SC4021E0, (**d**) subject SC4032E0.

In parallel, we also compared the original sample entropy values with the ones smoothed by Kalman filtering, moving average and EWMA for the four subjects respectively (Fig 5). Table 1 shows that VRR vaules are highest for Kalman filtering comparing to moving average and EWMA for most patients. Fig 6 shows that moving average takes more time than Kalman filtering or EWMA; while Kalman filtering and EWMA are close.

### EEG of CHB-MIT

**Fig 7:**
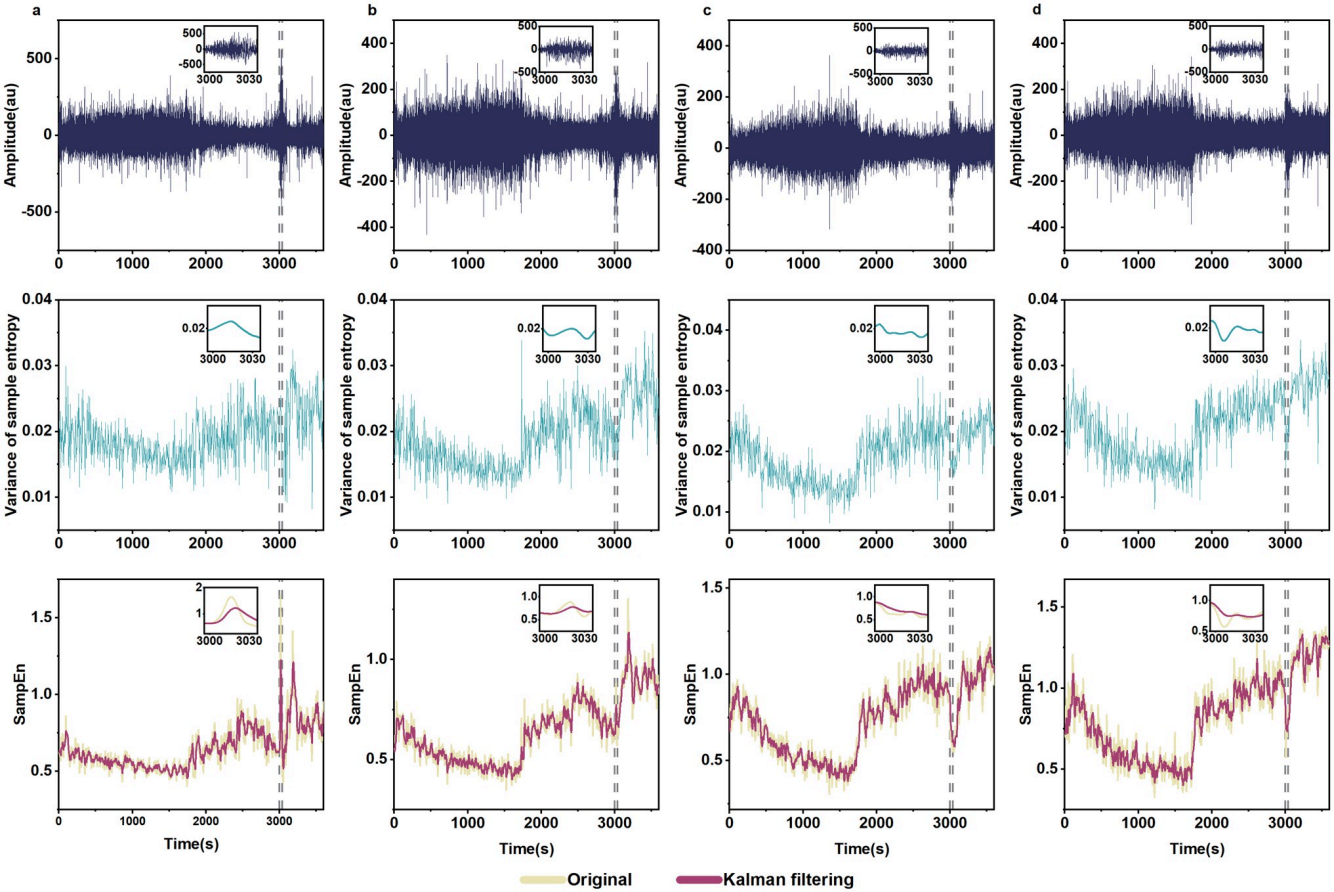
Kalman filtering on sample entropy time series of epilepsy signals. First row: waveforms of EEG recordings. Second row: theoretical variance of sample entropy calculated according to Eq 10. Third row: original and Kalman-filtering-smoothed sample entropy time series. (**a**): channel FP1-F3; (**b**): channel F3-C3; (**c**): channel C3-P3; (**d**): channel P3-O1.

EEG segments from four channels of a one-hour recording that contain seizures are illustrated in Fig 7, which are: Fp1-F3 (Fig 7a), F3-C3 (Fig 7b), C3-P3 (Fig 7c), and P3-O1 (Fig 7d). The seizure activities are highlighted in the insets (first row of Fig 7). The theoretical variance values of the sample entropy for the four channels were calculated according to Eq 10 (second row of Fig 7). Kalman filtering, moving average and EWMA reduce the variance of sample entropy for all channels (third row of Fig 7).

**Fig 8:**
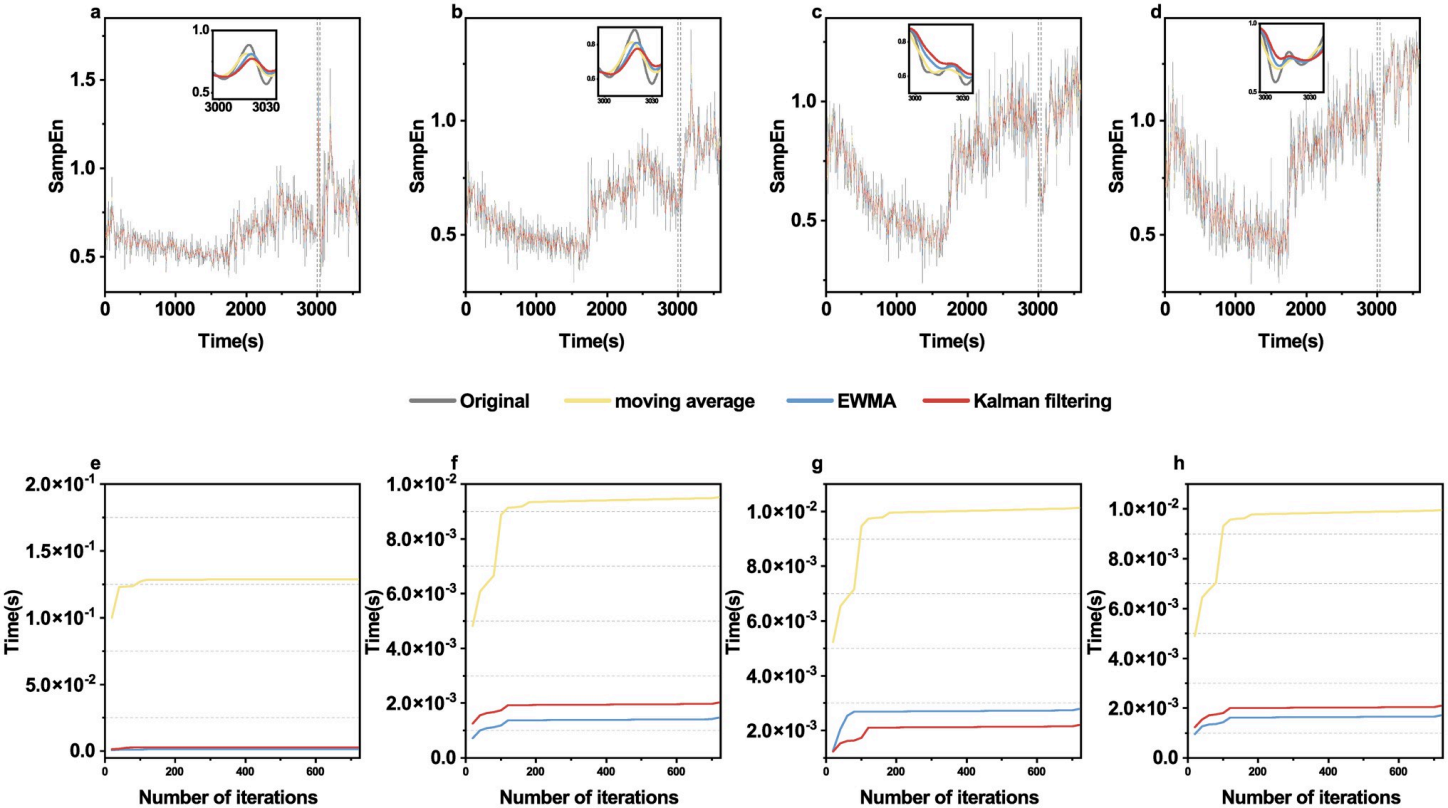
Comparison of the three smoothing methods for the epilepsy data. First row: original sample entropy time series and those smoothed by moving average, EWMA and Kalman filtering. Second row: cumulative computational time needed as a function of iteration number for channel FP1-F3 (**a,e**), channel F3-C3 (**b,f**), channel C3-P3 (**c,g**) and channel P3-O1 (**d,h**).

At the same time, we also compared entropy time series smoothed by Kalman filtering with the original sample entropy, as well as sample entropy smoothed by moving average and EWMA. As shown in Fig 8a–8d, moving average and EWMA moderately reduce the variance of the original entropy values, while Kalman filtering suppresses the variance to a larger extent. Table 2 shows highest VRR values by KF and lowest by MA.

**Table 2:** Comparison of VRR by different methods in for epilepsy data.

	FP1-F3	F3-C3	C3-P3	P3-O1
SampEn	2.52E-02	2.93E-02	4.77E-02	7.26E-02
SampEn after MA	1.97E-02	2.64E-02	4.21E-02	6.55E-02
SampEn after EWMA	1.91E-02	2.60E-02	4.20E-02	6.51E-02
SampEn after KF	1.75E-02	2.53E-02	4.08E-02	6.33E-02
VRR by MA	22.02%	9.99%	11.65%	9.89%
VRR by EWMA	24.19%	11.20%	11.89%	10.32%
VRR by KF	30.47%	13.77%	14.54%	12.86%

Note: SampEn stands for sample entropy; MA stands for moving average; KF stands for Kalman filtering.

In terms of time consumption, different brain regions show that Kalman filtering takes significantly less time than moving average and EWMA while moving average and EWMA are similar (Fig 8e–8h).

### Kalman filtering applied to other entropy measures

**Fig 9:**
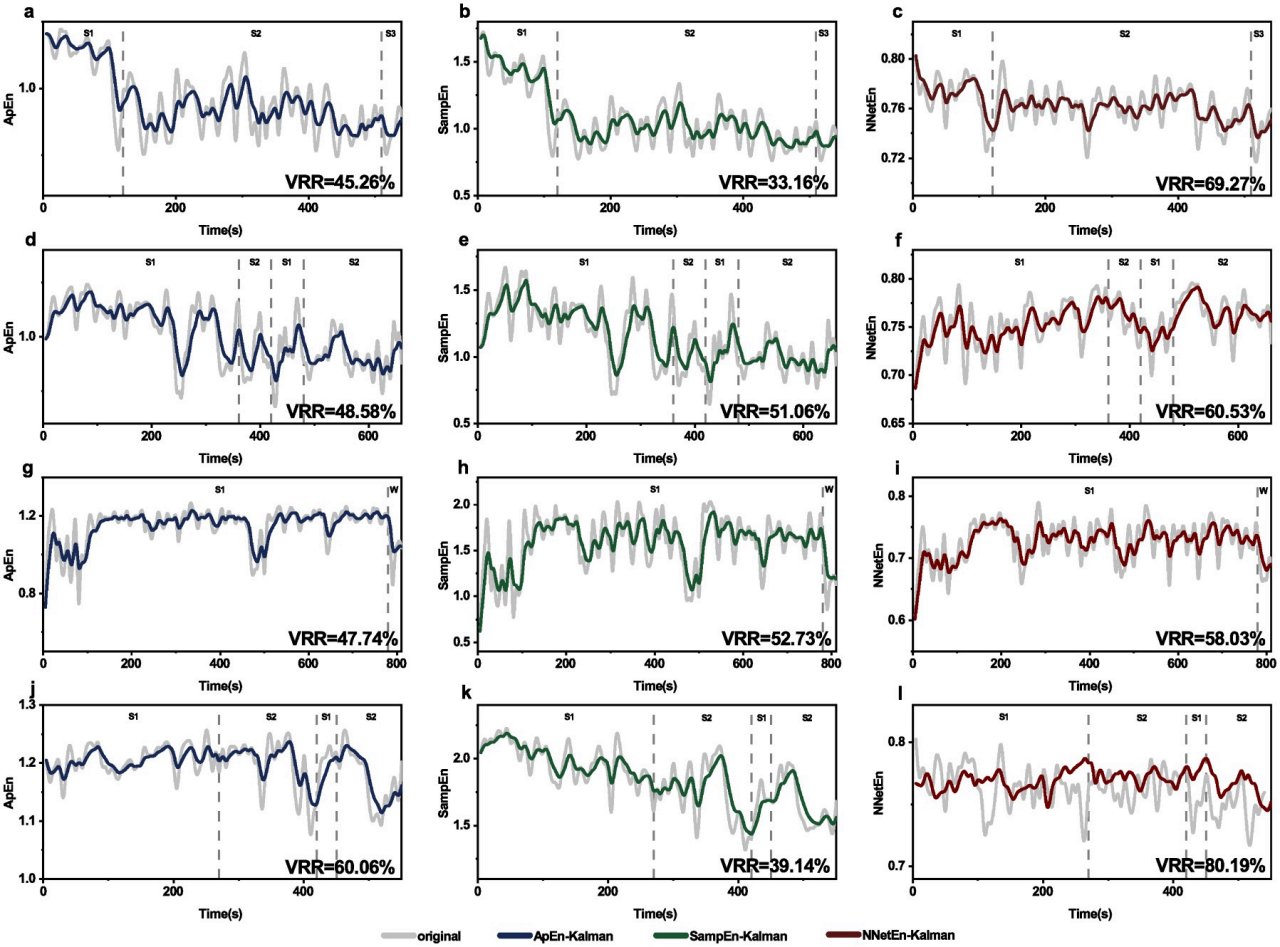
Kalman filtering applied to different entropy measures of sleep signals. Data are sleep signals from subjects SC4001E0 (**a,b,c**), SC4011E0 (**d,e,f**), SC4021E0 (**g,h,i**) and SC4032E0 (**j,k,l**). Kalman filtering is applied to approximate entropy (first row) and NNetEn (second row).

**Fig 10:**
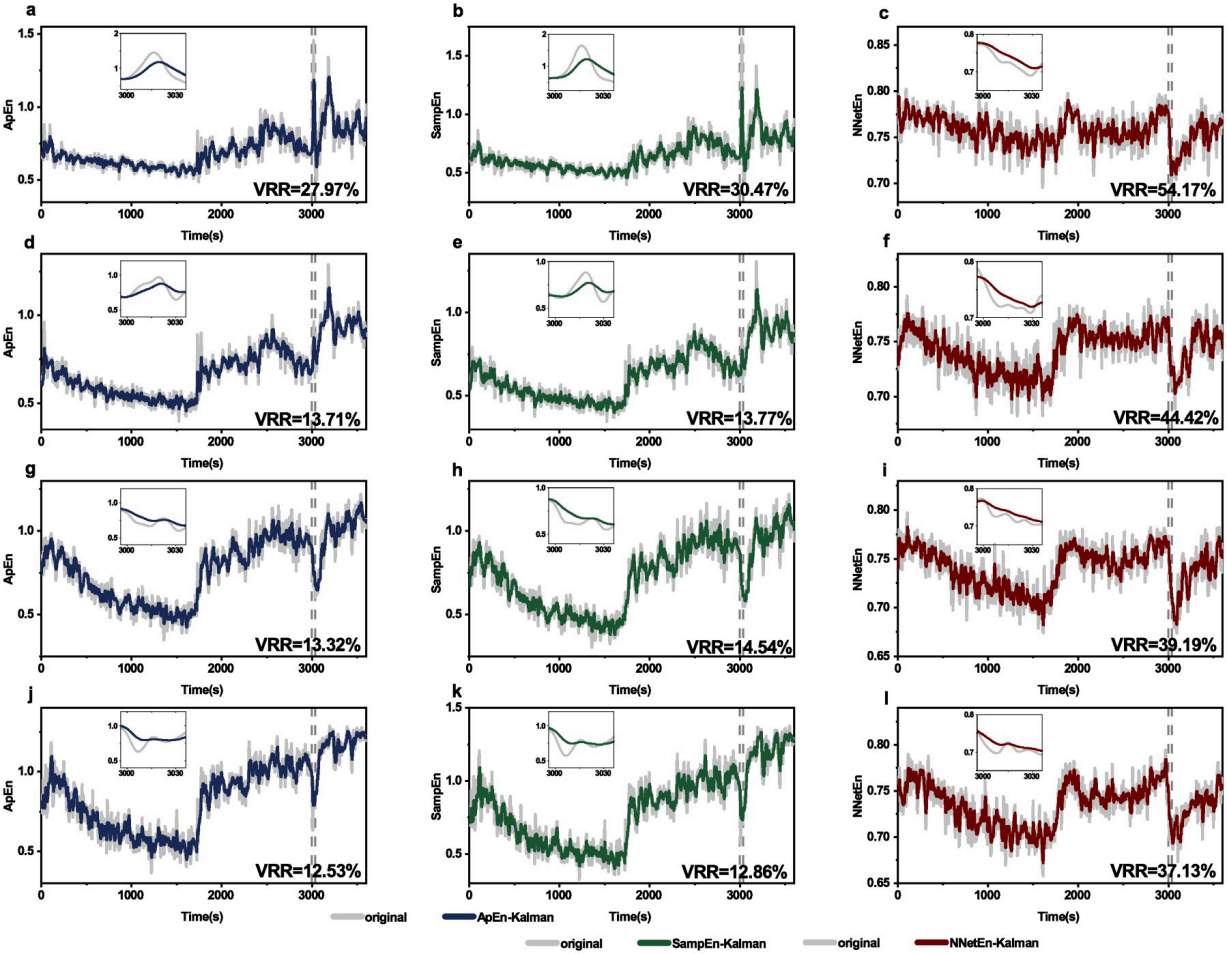
Kalman filtering applied to different entropy measures of epilepsy signals. Data are epilepsy signals from channel FP1-F3 (**a,b,c**), F3-C3 (**d,e,f**), C3-P3 (**g,h,l**) and P3-O1 (**j,k,l**). Kalman filtering is applied to approximate entropy (first row) and NNetEn entropy (second row).

Kalman filtering is applied to approximate entropy and NNetEn entropy for sleep signals (Fig 9) and epilepsy signals (Fig 10). VRR values for NNetEn entropy are higher than those for approximate entropy and sample entropy, indicating that NNetEn has lower measurement variance for both sleep and epilepsy signals.

### The effects of parameters for sample entropy

**Fig 11:**
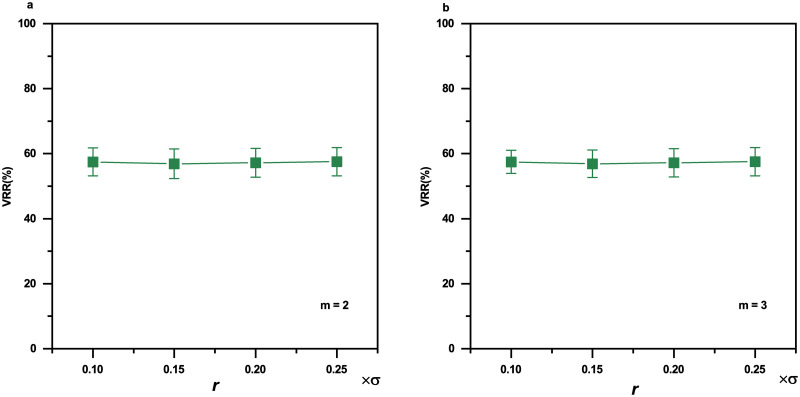
The effects of embedding dimension *m* and threshold *r* on VRR smoothed by Kalman filtering on sample entropy time series. (**a**)*m* = 2. (**b**)*m* = 3. The sleep EEG time series (10 subjects; data length of each subject: 50000 datapoints) were used for the sample entropy calculation. Error bars indicate standard errors. *σ* is the standard deviation of the EEG time series.

Fig 11 shows the effects of threshold *r* and embedding dimension *m* on VRR smoothed by Kalman filtering on sample entropy time series. For *m* = 2, the VRR values for *r* = 0.15, *r* = 0.15, *r* = 0.2 and *r* = 0.25 are not significantly different (*F* = 0.0038, *p* = 0.99967, one-way ANOVA). Similarly, for *m* = 3, the VRR values for *r* = 0.15, *r* = 0.15, *r* = 0.2 and *r* = 0.25 are not significantly different (*F* = 0.14755, *p* = 0.93059, one-way ANOVA).

**Fig 12:**
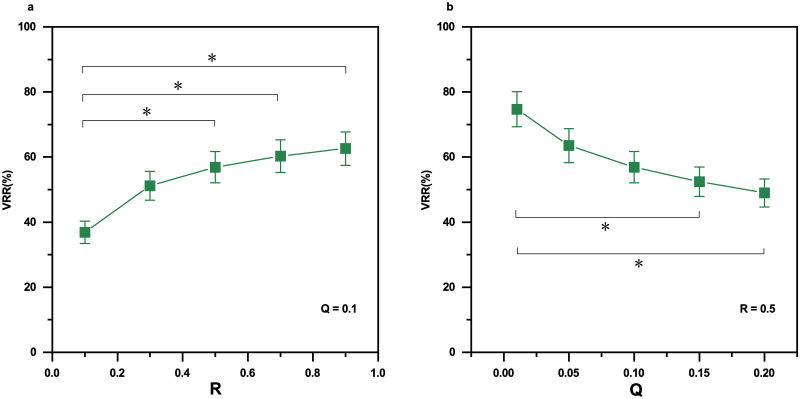
The effects of parameters *Q* and *P* on VRR smoothed by Kalman filtering on sample entropy time series. (a) VRR as a function of *R* values when *Q* is fixed to 0.1. (b) VRR as a function of *Q* values when *R* is fixed to 0.5. The data segment is the same as in Fig 11. Error bars indicate standard errors. N = 10. The sign * indicates significance level *p*<0.05.

Fig 12 shows the effects of hyperparameters *Q* and *R* on VRR smoothed by Kalman filtering on sample entropy time series. When *Q* is set to 0.1, five groups (*R* = 0.1, 0.3, 0.5, 0.7, 0.9) are significantly different (*F* = 4.93243, *p* = 0.0022, one-way ANOVA). When *R* is set to 0.5, five groups (*Q* = 0.01, 0.05, 0.10, 0.15, 0.20) are significantly different (*F* = 4.36334, *p* = 0.00457, one-way ANOVA).

## Discussion

Entropy is a measure of the amount of uncertainty associated with a variable [20]. Most studies on the entropy measurement of EEG focus on the values of entropy at some specific moments while in some circumstances, continuous monitoring of entropy is crucial, e.g., in monitoring depth of anesthesia [5], and epileptic EEG activity [10]. However, entropy as a measure suffers from the inherent noise, which is similar to a sensor’s measurement noise [27]. We have validated the feasibility that Kalman filtering can be used to smooth entropy time series obtained with a sliding window on simulated time series (power noise, Logistic map signals and Röslar system signals); see Figs 1–3. We have also applied Kalman filtering to sample entropy time series of sleep EEG (Fig 4) and epilepsy EEG (Fig 7) to show the smoothing effects.

To investigate the power of different smoothing methods, we have compared Kalman filtering to moving average, EWMA for sleep signals (Figs 5 and 6) and epilepsy data (Fig 8). The results show that VRR vaules are highest for Kalman filtering comparing to moving average and EWMA (Tables 1 and 2). Moving average and EWMA are commonly used with time series data to smooth out short-term fluctuations and highlight longer-term trends or cycles. There are other smoothing techniques, e.g., median average filtering or noise smoothing method based on wavelet thresholding techniques [50].

In addition to sample entropy, Kalman filtering can also be applied to other entropy measures. This is verified by two entropy measures as examples, one is a long-lasting entropy, i.e., approximate entropy [16], and the other is a newly invented one, i.e., NNetEn [47]. We have shown the applicability of Kalman filtering to these two entropy measures (Figs 9 and 10). VRR values for NNetEn entropy are higher than those for approximate entropy and sample entropy, indicating that NNetEn has lower measurement variance than approximate entropy for both sleep and epilepsy signals. We acknowledge that there exist a lot entropy measures (e.g., dispersion entropy [18], permutation entropy [51, 52], fuzzy entropy [53]), and expect a future exploration of applicability to other entropy measures.

We have set the transition matrix *A* and measurement matrix *H* to identity matrix. This is in accordance with the literature where, for the physiological time-series, the transition matrix is usually replaced by an identity matrix [54, 55].

For a typical selection of *m* = 2, there is no significant difference in the VRR of Kalman filtering in the range of *r* = 0.15 ∼ 0.25 (Fig 11). This means it is up to the users to choose *r* and we would suggest *r* = 0.15 to follow conventions [35, 45].

The two hyperparameters *Q* and *R* are covariances that can affect the performance of the Kalman filter (Fig 12). The process noise covariance *Q* reflexes the change of entropy measure. When *Q* is zero, there is no change at all for the entropy measure; on the other extreme, when *Q* is big enough, the entropy is allowed to change freely, which will lead to abrupt spikes. In this study, we have empirically set values of *Q* and *R*. We notice a series of work using adaptive methods in the determination of the two values [56]. In future work, we will explore adaptive methods to optimize these two hyperparameters.

Due to the limit of paper length, the synthetic signals used in this study are limited to power noise, Logistic map signals and Rösler signals. We note that there are some other synthetic signals that can be used for the evaluation of an algorithm, e.g., corrupted deterministic signal (MIX process) [6].

To our best knowledge, this is the first study that uses the Kalman filtering to track the change of entropy-based measures. The reason might be that the entropy-based measures are nonlinear methods that can quantify the rate of generation of new information [57], while at the same time, the Kalman filtering, in its initial form, is used for linear process. The rationale behind our proposal is that the entropy is a physical measure (despite of its complex nature) which may evolve with linear rules.

## Conclusion

Estimating entropy has been known to suffer from variance that arises from its calculation, producing measurement noise. We have for the first time addressed this issue and have used Kalman filtering technique to reduce the measurement noise. Kalman filtering is expected to be used to reduce measurement noise when continuous entropy estimation (for example anaesthesia monitoring) is essential with high accuracy and low time-consumption.

Database: https://doi.org/10.13026/C2X676.
